# From Decent Work to Decent Lives: Positive Self and Relational Management (PS&RM) in the Twenty-First Century

**DOI:** 10.3389/fpsyg.2016.00361

**Published:** 2016-03-23

**Authors:** Annamaria Di Fabio, Maureen E. Kenny

**Affiliations:** ^1^Department of Education and Psychology, University of FlorenceFlorence, Italy; ^2^Lynch School of Education - Boston CollegeChestnut Hill, MA, USA

**Keywords:** decent work, decent life, Positive Self and Relational Management (PS&RM), respectivity, relationality

## Abstract

The aim of the present study is to empirically test the theoretical model, Positive Self and Relational Management (PS&RM), for a sample of 184 Italian university students. The PS&RM model specifies the development of individuals' strengths, potentials, and talents across the lifespan and with regard to the dialect of self in relationship. PS&RM is defined theoretically by three constructs: Positive Lifelong Life Management, Positive Lifelong Self-Management, Positive Lifelong Relational Management. The three constructs are operationalized as follows: Positive Lifelong Life Management is measured by the Positive and Negative Affect Schedule (PANAS), the Satisfaction With Life Scale (SWLS), the Meaningful Life Measure (MLM), and the Authenticity Scale (AS); Positive Lifelong Self-Management is measured by the Intrapreneurial Self-Capital Scale (ISC), the Career Adapt-Abilities Scale (CAAS), and the Life Project Reflexivity Scale (LPRS); and Positive Lifelong Relational Management is measured by the Trait Emotional Intelligence Questionnaire (TEIQue), the Multidimensional Scale for Perceived Social Support (MSPSS), and the Positive Relational Management Scale (PRMS). Confirmatory factor analysis of the PS&RM model was completed using structural equation modeling. The theoretical PS&RM model was empirically tested as defined by the three hypothesized constructs. Empirical support for this model offers a framework for further research and the design of preventive interventions to promote decent work and decent lives in the twenty-first century.

## Introduction

Across the lifespan, individuals inevitably confront multiple challenges in the domains of work and relationship. While these are longstanding human challenges, recent economic, and social changes related to unemployment, globalization, and the changing nature of work have created a crisis involving underemployment, unemployment, and subsequent loss of financial security. The field of vocational psychology can assume a leadership role in responding to the current crisis by advancing theories, research, and practices that address the impact of these pressing social and economic challenges (Blustein, 2011b; Guichard, 2013b). While these challenges are experienced across all social classes, those who are poor and less educated are most vulnerable to the ill effects of social and economic change. Conditions of unemployment and employment that offers less than a living wage increase the number of persons living in poverty (Fryer and Fagan, 2003; Smith, 2010), along with the loss of psychological power (Prilleltensky, 2003) and heightened educational, health, and nutritional insecurities that accompany loss of material resources (Carr and Sloan, 2003; Sachs, 2005).

Vocational psychologists must seek to advance knowledge and services not only for the middle-class who exercise some degree of choice in their lives, but also for the poor and unemployed who typically have less volition with regard to their education and work lives (Blustein, 2006, 2011b). With few notable exceptions (Super, 1957; Saks, 2005), however, vocational psychology has not devoted significant attention to research and intervention for the poor or the unemployed (Blustein, 2011b). This study seeks to assess a model of Positive Self and Relational Management (PS&RM) intended to be applicable for promoting the strengths of individuals across varied social classes in meeting the economic and workplace challenges of the twenty-first century.

Current economic conditions are diminishing access to decent work for many segments of the population (ILO, 2015), while the salience of decent work as a fundamental human right central to human dignity and well-being has been widely affirmed. The concept of decent work was first introduced as part of the United Nations Universal Declaration of Human Rights (United Nations, 1948), which states that: “Everyone has the right to work, to the free choice of employment, to just and favorable conditions of work and to protection against unemployment; everyone, without any discrimination, has the right to equal pay for equal work; everyone who works has the right to just and favorable remuneration ensuring for himself and his family an existence worthy of human dignity, and supplemented, if necessary, by other means of social protection.” The Universal Declaration was confirmed by the International Labour Organization (ILO), the United Nations agency that aims to promote social justice and basic human and labor rights, stating that decent work should be assured for all workers (1999, 2015). Like the earlier UN proclamation, the ILO (1999, 2015) emphasized the importance of work availability for all those who want to work, basic rights for workers in the workplace (e.g., absence of discrimination or harassment and opportunities to exercise voice and participation through self-chosen representation), and working conditions that are safe, secure, and respect family and social values, provide a liveable income, and permit access to adequate health care.

The United Nations Educational, Scientific and Cultural Organization (UNESCO, 2009) has also reaffirmed the right for decent work, and the UNESCO Chair ≪Lifelong Guidance and Counselling≫ (Guichard, 2013a) has identified the in-depth study of decent work, including a more equitable approach to economic growth and sustainable opportunities for the working poor, as one of the challenges for the Chair (Guichard, 2013a; Guichard and Di Fabio, 2015). The Chair, founded at the University of Wroclaw, Poland in 2013, part of UNITWIN/UNESCO Chairs Program, facilitates the networking of scholars from internationally recognized world universities, to develop an integrated system of research, training, information and documented activities in educational and vocational counseling. The Chair moreover aims to foster equal opportunity, occupational mobility, and the development of vital career-related competencies through counseling projects and programs. The development of the PS&RM model was designed to meet the UNESCO call for research and intervention to develop personal competencies that will expand entry into decent work for the broad population.

In industrial and organizational psychology, the inclusive psychology of working (Blustein, 2006) offers a central theoretical framework relevant for conceptualizing the psychological importance of decent work for all people. According to this framework, decent work satisfies needs for power, relationship, and self-determination or the experience of engaging in activities that are experienced as authentic and motivating (Blustein, 2006). The psychology of working (Blustein, 2006) also recognizes that work and non-work experiences are highly interrelated, particularly in the sphere of human relationships. Blustein (2011a) enriched his theoretical articulation on the centrality of relationships in the relational theory of working, which recognizes that every work decision, experience, and interaction is influenced and shaped by relationships within and beyond the workplace (Blustein, 2011a). Decent work should thus foster competencies and conditions to support adaptive relationships in the workplace and beyond (Blustein, 2011a; Di Fabio, 2013a, 2014c). Moreover, given the importance of work and relationships as the major contexts of people's lives (Richardson, 2012), theory and practice need to be responsive to changes in contemporary life and guide people in constructing healthy lives through work and relationships. The PS&RM model affirms the role of work and relationships as interrelated and central to well-being for all people.

Additional theoretical shifts have emerged in vocational psychology in the twenty-first century in response to new challenges and transitions that have influenced and defined the PS&RM model. These theoretical developments include the shift from planning for career development to enhancing career management (Savickas, 2011b) and self-management skills (Guichard, 2013b; Di Fabio, 2014c). These theoretical developments are taking place in response to changes in work and life more broadly. While in the twentieth century individuals (at least those who were part of the broad middle class or above) were able to develop careers within a stable organization, career stability and security are less available for all in the twenty-first century (Savickas, 2011a). In the twenty-first century, work is often temporary and organizations are fluid (Savickas, 2011a). Rather that progressing through a predetermined and predictable series of stages (Super, 1957, 1980) or succession of vocational activities across the life span (Osipow, 1990), individuals now require a set of flexible career management and self-management skills to gain insight or reflexivity about themselves and their environments and to successfully navigate their increasingly unpredictable and chaotic career paths (Savickas, 2011b). For example, individuals have to maintain their employability when unemployed and actively manage their careers through adaptability, intentionality, life-long learning, autobiographical reasoning, and meaning-making (Savickas, 2011a). Life design is recognized as extending beyond career (Guichard, 2005) and integrating work and relationship in a dialectical manner across the entire life span (Di Fabio, 2014c). The notion of career management through self-management (Di Fabio, 2014c) thus emphasizes the importance of strengthening many aspects of self (Di Fabio, 2014b; Di Fabio et al., in press), including building resilience in order to adapt to changes in one's career and life pathways (Di Fabio et al., 2014; Di Fabio and Kenny, 2015). Career management and self-management draw upon psychological resources to maintain personal and social well-being in the midst of changing social and economic conditions and structures.

In the context of life design, the development of self and reflexivity are central processes (Savickas, 2011c; Guichard, 2013b). People are considered as having plural selves, with an individual's identity composed of a dynamic system of subjective identity forms (Guichard, 2005, 2013b). People interact in different contexts and with different experiences through which they develop different images of the self and take on roles that are different from one context to another (Guichard, 2005, 2013b). Through a dynamic and continuous process of reflexivity, individuals expand self-awareness and attribute meaning to their life experiences (Savickas, 2011a; Guichard, 2013b; Bernaud, 2015). In counseling dialogues, reflexivity enables individuals to discover their complexity and plurality, create personal meaning, define future objectives, and construct a future self with purpose and authenticity (Guichard, 2004, 2005; Di Fabio, 2014f).

In addition to shifts in vocational theory, the PS&RM model builds on developments in positive youth development (PYD, Catalano et al., 2004; Lerner et al., 2005; Di Fabio et al., 2014; Kozan et al., 2014) and positive psychology (Seligman and Csikszentmihalyi, 2000; Seligman, 2002), with a focus on the attainment of well-being from hedonic and eudaimonic perpsectives. Hedonic well-being refers to pleasure attainment and pain avoidance (Kahneman et al., 1999), while eudaimonic well-being refers to an optimal state of functioning, focusing on the cultivation of resources and strengths, personal self-realization, and the attainment of personal meaning and pursuing life according to the true or authentic self (Ryan and Deci, 2001; Vázquez et al., 2006; Ryff and Singer, 2008; Waterman et al., 2010). Whereas hedonic and eudaimonic well-being are both valuable, eudaimonic well-being is particularly relevant to lifelong positive development. The pursuit of happiness or pleasure can lead persons to embrace goals and engage in activities that are not ultimately aligned with the realization of one's full potential or of benefit to society (Ryan and Deci, 2001; Ryff and Singer, 2008).

PYD emphasizes the importance of building individual resources and strengths, not only as protective factors for coping with challenge, but as resources that allow youth to fully thrive and contribute meaningfully to society (Di Fabio et al., in press). Positive Adult Development (PAD; Helson and Srivastava, 2001; Commons, 2002) similarly emphasizes the capacity of individuals to cope adaptively with change and challenge across the life course (Helson and Srivastava, 2001; Commons, 2002). Positive Lifelong Development (PLD; Colby and Damon, 1992) highlights resources that contribute to optimal health and life quality (Colby and Damon, 1992). In consideration of the above frameworks, PS&RM specifies positively lifelong development as “the development of individuals' strengths, potentials, and varied talents from a lifespan perspective and the positive dialectic of the self in relationship” (Di Fabio, 2014b). The PS&RM model focuses on the promotion of self and relational management across varied personal and professional transitions toward the attainment of identitarian purposeful awareness and positive lifelong development (Di Fabio, 2014f). Identitarian purposeful awareness entails the realization of the authentic self, accompanied by a sense of meaning and purpose (Di Fabio, 2014f), and is thus congruent with eudaimonic conceptions of well-being.

In sum, the PS&RM model (Di Fabio, 2014b, 2015a) model was formulated for addressing the complex work and life challenges of the twenty-first century (Di Fabio, 2014b, 2015a) and the integral dialect of relationship across work and life contexts (Guichard, 2004; Blustein, 2011a; Savickas, 2011a; Di Fabio, 2014b, 2015a). PS&RM adopts a preventive perspective (Hage et al., 2007; Kenny and Hage, 2009; Di Fabio et al., in press), underlining the importance of individual strengths (Di Fabio and Palazzeschi, 2008a,b, 2009, 2012; Di Fabio and Blustein, 2010; Di Fabio and Kenny, 2012a; Di Fabio et al., 2012, 2013, 2014; Di Fabio and Saklofske, 2014a,b) and relational strengths (Blustein, 2011a; Di Fabio and Kenny, 2012b) to face the challenges of the twenty-first century and promote overall-well-being. PS&RM embraces Positive Psychology (Seligman and Csikszentmihalyi, 2000; Seligman, 2002), is aligned with PYD (Lerner, 2002; Kenny, 2007), and is informed by the inclusive psychology of working (Blustein, 2006), the relational theory of working (Blustein, 2011a), and contemporary developments in career theory, including Career Construction (Savickas, 2005), Self-Construction and Life-Construction (Guichard, 2005, 2013b), Life Meaning (Bernaud, 2015), and meta-reflection and reflexivity emphasizing self-insight and awareness (Guichard, 2009, 2013b; Maree, 2013; Di Fabio, 2014c).

At a more specific level, the PS&RM theoretical model is defined by three constructs: Positive Lifelong Life Management, Positive Lifelong Self-Management, and Positive Lifelong Relational Management. The operationalization of this model is as follows. The first construct, Positive Lifelong Life Management encompasses hedonic and eudaimonic well-being, and is measured by the Positive and Negative Affect Schedule (PANAS, Watson et al., 1988) and the Satisfaction With Life Scale (SWLS, Diener et al., 1985), as indices of hedonic well-being; by Meaningful Life Measure (MLM, Morgan and Farsides, 2009) and the Authenticity Scale (AS; Wood et al., 2008), as indices of eudaimonic well-being, emphasizing life meaning and authenticity. The second construct, Positive Lifelong Self-Management emphasizes individual level resources and self-insight for coping adaptively with change in the work domain and is assessed by Intrapreneurial Self-Capital Scale (ISC; Di Fabio, 2014d), the Career Adapt-Abilities Inventory (Savickas and Porfeli, 2012), and the Life Project Reflexivity Scale (Di Fabio, 2015b). The third construct, Positive Lifelong Relational Management assesses resources for relational adaptation within the workplace and beyond and is operationalized by the Trait Emotional Intelligence Questionnaire (TEIQue; Petrides and Furnham, 2004), the Multidimensional Scale of Perceived Social Support (MSPSS; Zimet et al., 1988), and the Positive Relational Management Scale (Di Fabio, 2015c).

The operationalization of the three PS&RM constructs is based upon a significant body of existing research. The measures of Positive Lifelong Life Management are widely used assessments of hedonic and eudaimonic well-being, including life meaning and authenticity, and have been associated with a range of valued life outcomes for individuals and for society (Ryan and Deci, 2001). Positive Lifelong Self-Management incorporates well-known constructs and measures, such as the Career Adapt-Abilities Inventory, developed, and studied by vocational psychologists interested in the individual resources needed for adapting to an uncertain and changing career context (Porfeli and Savickas, 2012; Savickas and Porfeli, 2012). Intrapreneurial self-capital (ISC) was developed by Di Fabio (2014c) to further specify and assess skills relevant for innovative and effective problem-solving in the context of uncertainty and change. The ISC metaconstruct encompasses nine individual factors (e.g., hardiness, resilience, positive self-concept, creative self-efficacy, decisiveness, and goal mastery) found in prior research to be associated with career adaptability, employability, and well-being (Di Fabio, 2014b). Di Fabio (2014c) confirmed the factor structure of the ISC metaconstruct as derived from the seven underlying constructs. The new ISC scale was found to correlate positively with career decision-self efficacy, perceived employability and school grade point average (GPA), and negatively with career decision-making difficulties. The Positive Lifelong Relational Management construct similarly incorporates widely used relational and social skills measures, such as emotional intelligence and social support, as well as a measure developed specifically to assess competencies relevant to the current challenges. The new Positive Relational Management Scale (PRMS, Di Fabio, 2015c) focuses on three positive dimensions of relationships, respect, care, and connection, that are relevant across multiple life domains.

The aim of this study is to empirically test the PS&RM theoretical model. Specifically, the present study addresses the following question: is there empirical evidence for the conceptualization of PS&RM as constituted by the three constructs of Positive Lifelong Life Management, Positive Lifelong Self-Management, Positive Lifelong Relational Management? In other words, does evidence exist for the latent construct PS&RM operationalized by the three lower order latent constructs of Positive Lifelong Life Management, Positive Lifelong Self-Management, and Positive Lifelong Relational Management?

## Materials and methods

### Participants

Participants are 184 Italian students of the University of Florence. Regarding gender, 81 participants were male (44%) and 103 participants were female (56%). The age of the participants ranged from 22 to 27 years (*M* = 24.13; *SD* = 1.79). Participants were predominantly White Italians from middle-class backgrounds.

### Measures

#### Positive and negative affect schedule (PANAS)

To evaluate positive affect (PA) and negative affect (NA), the Positive and Negative Affect Schedule (PANAS, Watson et al., 1988) in the Italian version by Terracciano et al. (2003) was used. The PANAS is composed of 20 adjectives of which 10 refer to Positive Affect (PA; e.g., enthusiastic, interested, determined) and 10 to Negative Affect (NA; e.g., afraid, upset, distressed). The participants has to indicate to what extent they generally feel on average on a Likert scale from 1 = Very slightly or not at all to 5 = Extremely. The Cronbach's alpha coefficients were:0.71 for Positive Affect and 0.81 for Negative Affect in the present study;0.72 for Positive Affect and 0.83 for Negative Affect in the Terracciano et al. (2003) study. Regarding the validity of the Italian version of the PANAS, a positive but moderate relation was found between Positive Affect and Extraversion and an inverse but moderate relation was found between Negative Affect and Neuroticism, similar to findings with the original American version (Terracciano et al., 2003). Furthermore the Italian version of the PANAS showed predictive validity in relation to depression (Terracciano et al., 2003).

#### Satisfaction with life scale (SWLS)

To evaluate life satisfaction, the Satisfaction With Life Scale (SWLS, Diener et al., 1985) in the Italian version by Di Fabio and Gori (2015) was used. The scale consist of five items (e.g., “I am satisfied with my life,” “The conditions of my life are excellent”) on a 7-point Likert scale from 1 = Strongly disagree to 7 = Strongly agree. The scale has a one-dimensional factorial structure. The Cronbach's alpha coefficient is 0.88 in the present study and 0.85 in the Di Fabio and Gori (2015) study that validated the Italian version of the scale. Regarding concurrent validity, Di Fabio and Gori found the Italian version of the SWLS to be positively correlated with the Rosenberg Self-Esteem Scale (Rosenberg, 1965).

#### Meaningful life measure (MLM)

To evaluate meaning in life, the Meaningful Life Measure (MLM, Morgan and Farsides, 2009) in the Italian version by Di Fabio (2014e) was used. The questionnaire is composed of 23 items on a 7-point Likert scale from 1 = Strongly disagree to 7 = Strongly agree. The MLM detects five dimensions: Exciting life (e.g., “Life to me seems always exciting”), Accomplished life (e.g., “So far, I am pleased with what I have achieved in life”), Principled life (e.g., “I have a personal value system that makes my life worthwhile”), Purposeful life (e.g., “I have a clear idea of what my future goals and aims are”), Valued life (e.g., “My life is significant”). The Cronbach's alpha coefficients were: for Exciting life, 0.85 in the present study and 0.87 in the study relative to the Italian version; for Accomplished life 0.83 in the present study and 0.86 in the Di Fabio (2014e) study that validated the scale for the Italian context; for Principled life, 0.84 in the present study and 0.85 in the Italian validation study; for Purposeful life, 0.84 in the present study and 0.87 in the Italian validation; for Valued life, 0.83 in the present study and 0.85 for the Italian validation. Regarding the concurrent validity of the Italian version of the MLM, positive relationships emerged with life satisfaction and positive affect and inverse relationships with negative affect (Di Fabio, 2014e).

#### Authenticity scale (AS)

To evaluate authenticity, the Authenticity Scale (AS; Wood et al., 2008) in the Italian version by Di Fabio (2014a) was used. The measure is composed of 12 items with a response format on a 7-point Likert scale ranging from 1 = Does not describe me at all to 7 = Describes me very well. The scale provides a total score of authenticity and also the scores for three dimensions of authenticity: Self-alienation (e.g., “I feel out of touch with the real me”), Authentic living (e.g., “I am true to myself in most situations”), and Accepting external influence (e.g., “I always feel I need to do what others expect of me”). The Cronbach's alpha coefficients were: for Self-alienation, 0.83 in the present study and 0.81 in the Di Fabio (2014a) study that validated the Italian version; for Authentic living, 0.80 in the present study and 0.79 in the Italian validation study; for Accepting external influence, 0.81 in the present study and 0.84 in the Italian validation study. Regarding concurrent validity of the Italian version of the AS, positive relationships emerged with self-esteem, life satisfaction, and positive affect and inverse relationships with negative affect (Di Fabio, 2014a).

#### Intrapreneurial self-capital scale (ISC)

The Intrapreneurial Self-Capital Scale (ISC; Di Fabio, 2014c) was used to assess a higher order construct related to the individual resources for innovation and problem solving in the context of uncertainty. The ISC scale is composed of 28 items (e.g., “I am able to deal with most of my problems,” “I'm able to improve the ideas produced by others,” “One of my goals in training is to learn as much as I can”) with a response format on a 5-point Likert scale ranging from 1 = Strongly agree to 5 = Strongly disagree. The Cronbach's alpha coefficient was 0.84 in the present study and 0.86 in the Di Fabio (2014c) study reporting on the development of the scale. Regarding the validity of the ISC, a positive relationship between ISC and academic performance in terms of Grade Point Average supported predictive validity of the scale (Di Fabio, 2014c). Furthermore the positive relationship of the ISC with perceived employability and career decision self-efficacy and the inverse relation of ISC with career decision-making difficulties supported concurrent validity of the scale (Di Fabio, 2014c).

#### Career adapt-abilities scale (CAAS)

To evaluate career adaptability, the Career Adapt-Abilities Scale (CAAS; Savickas and Porfeli, 2012) in the Italian version for young adults (Di Fabio, 2016c) was used. The scale is composed of 24 items with a response format on a 5-point Likert scale ranging from 1 = Strongest to 5 = Not strong. The scale assesses four dimensions of career adaptability: Concern (e.g., “Thinking about what my future will be like,” “Realizing that today's choices shape my future”), Control (e.g., “Taking responsibility for my actions,” “Doing what's right for me”), Curiosity (e.g., “Looking for opportunities to grow as a person,” “Becoming curious about new opportunities”), Confidence (e.g., “Performing tasks efficiently,” “Working up to my ability”). The Cronbach's alpha coefficients were: for Concern, 0.83 in the present study and 0.85 in the Di Fabio (2016c) study that validated the Italian version for young adults; for Control, 0.84 in the present study and 0.86 in the Di Fabio (2016c) study; for Curiosity, 0.82 in the present study and 0.83 in the Di Fabio (2016c) study; for Confidence, 0.81 in the present study and 0.82 in the study relative to the Italian version for young adults. Regarding the Italian version of the CAAS for young adults, relationships emerged between the CAAS and both self-perceived employability and authenticity (Di Fabio, 2016c).

#### Life project reflexivity scale (LPRS)

The Life Project Reflexivity Scale (LPRS; Di Fabio, 2015b) was used to assess reflexivity on one's future career and life projects according to the authentic self, as opposed to acquiescing to others. The scale is composed of 21 items and three dimensions (e.g., Project actuality: “The projects for my future life are clear and defined”; Authenticity: “The projects for my future life are aligned with my most authentic values”; Acquiescence: “The projects for my future life are aligned with societal values, rather than with my most authentic values”) with a response format on a 5-point Likert scale ranging from 1 = Strongly agree to 5 = Strongly disagree. The Cronbach's alpha coefficients were: for Project actuality, 0.76 in the present study and 0.77 in the scale development study (Di Fabio, 2015b); for Authenticity, 0.79 in the present study and 0.78 in the Di Fabio (2015b) study; for Acquiescence, 0.82 in the present study and 0.80 reported by Di Fabio (2015b). Regarding the concurrent validity, positive relationships emerged between the LPRS and both authenticity and meaning in life (Di Fabio, 2015b).

#### Trait emotional intelligence questionnaire (TEIQue)

To evaluate trait emotional intelligence, the Trait Emotional Intelligence Questionnaire (TEIQue; Petrides and Furnham, 2004) in the Italian version by Di Fabio (2013b) was used. The questionnaire is composed of 153 items with response options on a 7-point Likert scale format ranging from 1 = Completely disagree to 7 = Completely agree. The questionnaire provides a total score, and scores for four principal dimensions: Well-being (e.g., “I feel that I have a number of good qualities,” “On the whole, I'm pleased with my life,” “I generally believe that things will work out fine in my life”), Self-Control (e.g., “I'm usually able to calm down quickly after I've got mad at someone,” “I would describe myself as a calm person,” “Controlling my urges is not a big problem for me”), Emotionality (e.g., “I often find it difficult to recognize what emotion I'm feeling,” “I find it difficult to tell others that I love them even when I want to,” “It is very important to me to get along with all my close friends and family”), and Sociability (e.g., “I can deal effectively with people,” “If I wanted to, it would be easy for me to make someone angry,” “I have many reasons for not giving up easily”). The Cronbach's alpha coefficients were: for Well-being 0.88 in the present study and 0.90 in the Di Fabio (2013b) study that developed the Italian version; for Self-control, 0.82 in the present study and 0.83 in the Di Fabio (2013b) study;0.83 in the present study and 0.86 in Di Fabio (2013b); for Sociability, 0.82 in the present study and 0.85 in Di Fabio (2013b). Regarding convergent and discriminant validity of the Italian version of the TEIQue (Di Fabio, 2013b), positive correlations emerged between TEIQue and Bar-On Emotional Quotient Inventory (Bar-On EQ-I, Bar-On, 1997). In contrast, low and non-significant correlations between the TEIQue and ability-based emotional intelligence detected through the Mayer, Salovey and Caruso Emotional Intelligence Test (MSCEIT, Mayer et al., 2002), indicating that these measures tap two different aspects of the same construct. Furthermore, low to moderate positive correlations between the TEIQue and personality traits emerged, indicating that Trait EI presents some overlap with aspects of personality, but is also a distinct construct.

#### Multidimensional scale for perceived social support (MSPSS)

The total score of Multidimensional Scale for Perceived Social Support (MSPSS; Zimet et al., 1988) in the Italian version by Di Fabio and Palazzeschi (2015) was used to evaluate perceived social support from family, friends, and significant others. The scale is composed of 12 items (e.g., “My family works very hard to help me,” “I can speak about my problems with my friends,” “When I need someone, there is always a special person who stands by me,” with response options in a 7-point Likert scale format ranging from 1 = Strongly disagree to 7 = Strongly agree. The Cronbach's alpha coefficients were: for family support, 0.91 in the present study and 0.92 in the Di Fabio and Palazzeschi (2015) study that validated the Italian version; for friends support, 0.94 in the present study and 0.90 in Di Fabio and Palazzeschi (2015); for the significant others support, 0.90 in the present study and 0.93 in the Di Fabio and Palazzeschi (2015) study. Regarding concurrent validity, positive relationships emerged between the MSPSS, and social support (Di Fabio and Palazzeschi, 2015).

#### Positive relational management scale (PRMS)

The Positive Relational Management Scale (PRMS; Di Fabio, 2015c) assesses three positive dimensions of relationships, including respect (my respect for others, respect of others for myself, my respect for myself), care (my care for others, care of others for myself, my care for myself), and connection (my connection with family, with friends, with significant others). The scale is composed of 12 items and three dimensions (e.g., Respect: “I keep a balance between respect toward others and toward myself”; Caring: “I often take care of others”; Connection: “I have good relationships with my family”) with a response format on a 5-point Likert scale ranging from 1 = Strongly agree to 5 = Strongly disagree. The Cronbach's alpha coefficients were: for Respect 0.80 in the present study and 0.81 in the Di Fabio (2015c) study reporting on the development of the scale; for Caring, 0.80 in the present study and 0.79 in the Di Fabio (2015c) study; for Connection, 0.81 in the present study and 0.80 in Di Fabio (2015c). Regarding the concurrent validity, positive relationships emerged between the PRMS and perceived social support (Di Fabio, 2015c).

### Procedure and data analysis

The questionnaires were administered to groups of participants by trained psychologists. Students participated voluntarily in the study and were not compensated. The order of administration of the questionnaires were counterbalanced to control for possible effects of presentation during the administration. The questionnaires were administered according to the laws of privacy and informed consent of the Italian law (Law Decree DL-196/2003). Concerning ethical standards for research, the study followed procedures consistent with the latest version of the Declaration of Helsinki revised in Fortaleza [World Medical Association World Medical Association (WMA), 2013].

Descriptive statistics and Pearson's r correlations were computed. Confirmatory factor analysis of the PS&RM model conducted through structural equation modeling using AMOS version 6 (Arbuckle, 2005). The adequacy of the model was tested based upon the χ^2^/df, the Comparative Fit Index (CFI, Bentler, 1990) and the Non-Normed Fit Index (NNFI, Tucker and Lewis, 1973), and the Root Mean Square Error of Approximation (RMSEA, Browne and Cudeck, 1993).

## Results

Means, standard deviations, and correlations between the variables included in the model are reported in Table 1.

**Table 1:** **Means, standard deviations, and correlations between the variables included in**.

	***M***	***DS***	**1**	**2**	**3**	**4**	**5**	**6**	**7**	**8**	**9**	**10**	**11**	**12**	**13**	**14**	**15**	**16**	**17**
1. Positive Affect	20.12	6.13	−																
2. Negative Affect	20.19	5.35	0.76^***^	−															
3. Life satisfaction	21.65	5.97	0.57^**^	−0.45^**^	−														
4. Meaning in life	92.40	14.17	0.40^**^	−0.33^**^	0.43^**^	−													
5. Authenticity	44.60	7.48	0.32^**^	−0.28^*^	0.47^**^	0.52^**^	−												
6. ISC	101.54	10.24	0.49^**^	−0.48^**^	0.43^**^	0.57^**^	0.37^**^	−											
7. Concern	19.69	4.65	0.30^**^	−0.29^*^	0.35^**^	0.33^**^	0.13	0.36^**^	−										
8. Control	20.54	4.52	0.26^**^	−0.24^*^	0.39^**^	0.37^**^	0.23^*^	0.45^**^	0.44^**^	−									
9. Curiosity	21.28	4.14	0.29^**^	−0.24^*^	0.33^**^	0.30^**^	0.12	0.34^**^	0.36^**^	0.49^**^	−								
10. Confidence	21.86	4.54	0.30^**^	−0.22^*^	0.37^**^	0.32^**^	0.23^**^	0.50^**^	0.41^**^	0.68^**^	0.56^**^	−							
11. LPRS	70.88	10.13	0.32^**^	−0.29^**^	0.36^**^	0.37^**^	0.20^*^	0.45^**^	0.28^**^	0.42^**^	0.19^*^	0.29^**^	−						
12. Well-being	15.10	4.81	0.48^**^	−0.41^**^	0.44^**^	0.37^**^	0.37^**^	0.61^**^	0.21^*^	0.40^**^	0.19^*^	0.39^**^	0.44^**^	−					
13. Self-control	14.20	4.61	0.22^*^	−0.21^**^	0.46^**^	0.39^**^	0.25^**^	0.40^**^	0.17	0.25^**^	0.07	0.28^**^	0.20^**^	0.25^**^	−				
14. Emotionality	15.00	4.70	0.33^**^	−0.31^**^	0.44^**^	0.40^**^	0.47^**^	0.37^**^	0.23^**^	0.32^**^	0.30^**^	0.23^*^	0.25^**^	0.44^**^	0.30^**^	−			
15. Sociability	14.61	4.60	0.35^**^	−0.33^**^	0.48^**^	0.38^**^	0.41^**^	0.50^**^	0.29^**^	0.29^**^	0.26^**^	0.34^**^	0.19^*^	0.51^**^	0.16	0.50^**^	−		
16. MSPSS	23.29	4.65	0.26^**^	−0.30^**^	0.28^**^	0.25^**^	0.19^*^	0.12	0.13	0.10	0.03	0.12	0.16	0.24^**^	0.05	0.14	0.16	−	
17. PRSM	47.33	5.06	0.39^**^	−0.35^**^	0.40^**^	0.36^**^	0.28^**^	0.34^**^	0.13	0.24^**^	0.23^**^	0.20^*^	0.33^**^	0.46^**^	0.14	0.33^**^	0.41^**^	0.20*	−

*N = 184*.

* p < 0.05;

** p < 0.01;

*** *p < 0.001*.

**Figure 1:**
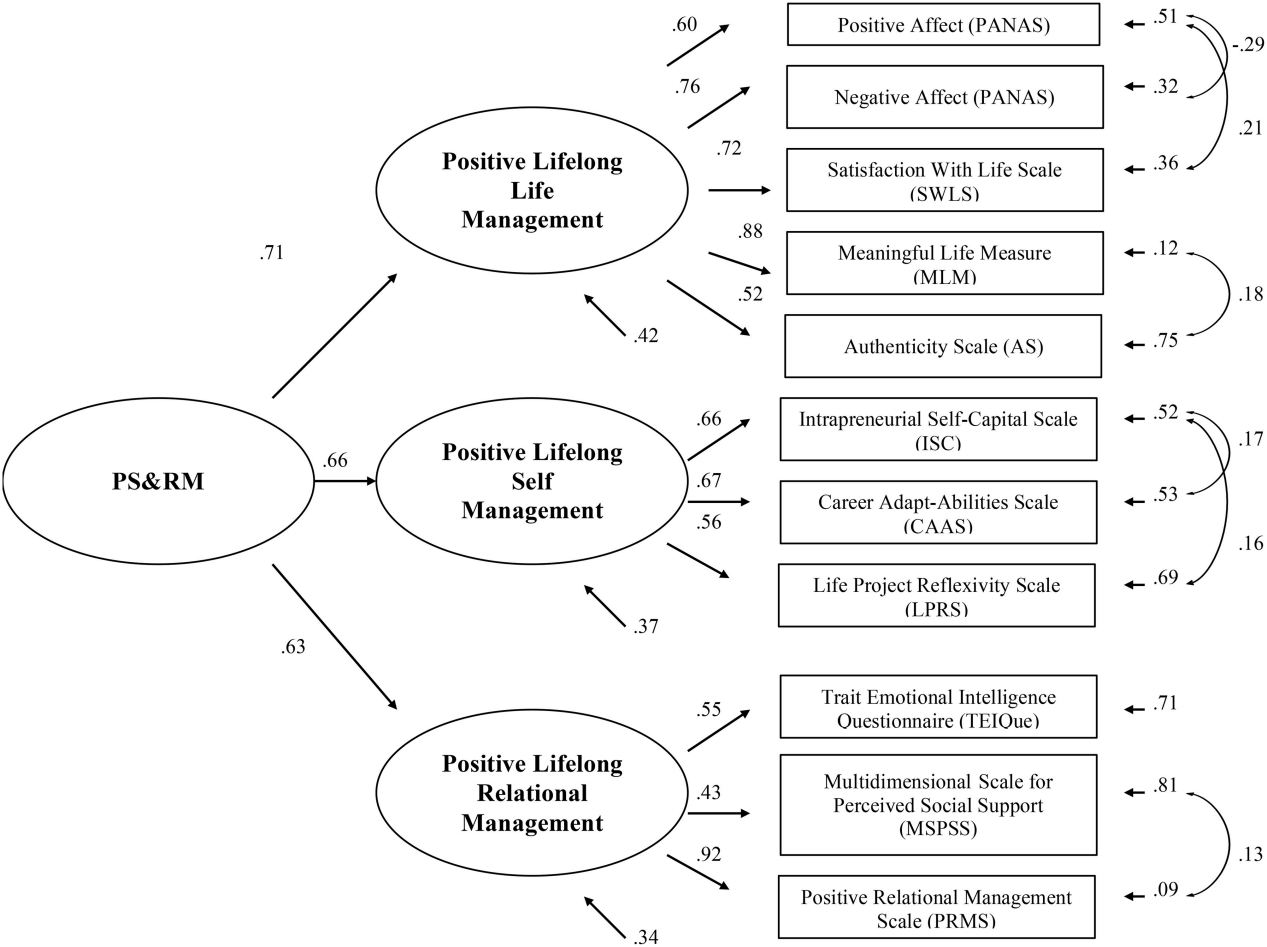
**PS&RM model**.

To test the PS&RM model through confirmatory factor analysis, structural equation modeling was conducted using the total scores for each variable included as an oberved variables in the model. The observed variables as previsouly described include: positive affect and negative affect (PANAS, Watson et al., 1988), life satisfaction (SWLS, Diener et al., 1985), meaning in life (MLM, Morgan and Farsides, 2009) and authenticity (Wood et al., 2008) to assess Positive Lifelong Life Management; Intrapreneurial Self-Capital (ISC; Di Fabio, 2014d), career adaptability (Concern, Control, Curiosity, Confidence; Savickas and Porfeli, 2012), and life project reflexivity (Di Fabio, 2015b) to assess Positive Lifelong Self-Management; and the four TEIQue dimensions (Well-being, Self-Control, Emotionality, Sociability; Petrides and Furnham, 2004), perceived social support (Zimet et al., 1988), and positive relational management (Di Fabio, 2015c) to assess Positive Lifelong Relational Management. Goodness of fit for the model was evaluated using established criteria (See Figure 1). Values of the χ^2^/df between 1 and 3 are considered indices of a good model fit. The Comparative Fit Index (CFI, Bentler, 1990) and the Non-Normed Fit Index (NNFI, Tucker and Lewis, 1973) are considered indicative of a good fit when the values are higher than 0.90 (Bentler, 1990). Also values for the Root Mean Square Error of Approximation (RMSEA, Browne and Cudeck, 1993) lower than 0.08 also indicate a good model fit. In Table 2 fit indices and fit changes for different compared models are reported. The first model corresponds to the theoretical hypothesized model with a latent construct PS&RM operationalized by three lower order latent constructs: Positive Lifelong Life Management with five indicators, Positive Lifelong Self-Management with three indicators, and Positive Lifelong Relational Management with three indicators. Inspection of the modification indices suggested several changes to improve the model fit. Considering that the correlations among the indicators, as apparent in Table 1 and theoretical support for these correlations, subsequent models allowed residual errors among the indicators to correlate. As such, we inserted one covariance (starting from the more significant) in each model, with the final model showing covariance of indicators within model constructs.

**Table 2:** **Fit indices and fit changes for model 1 trough model 7**.

	**χ^2^ (df)**	**RMSEA**	**CFI**	**NNFI**	**Δ χ^2^(Δdf)**	**Δ Sig**.
Model 1	59.42(43)	0.11 CI [0.10, 0.13]	0.89	0.89		*p* < 0.05
Model 2	58.34(42)	0.10 CI [0.09, 0.11]	0.89	0.90	1.08(1)	*p* < 0.05
Model 3	55.74(41)	0.09 CI [0.08, 0.10]	0.90	0.90	2.60(1)	*p* < 0.05
Model 4	54.37(40)	0.09 CI [0.07, 0.10]	0.91	0.90	1.37(1)	*p* < 0.05
Model 5	52.73(39)	0.08 CI [0.06, 0.09]	0.91	0.91	1.64(1)	*p* < 0.05
Model 6	50.54(38)	0.07 CI [0.06, 0.08]	0.92	0.91	2.19(1)	*p* < 0.05
Model 7	44.77(37)	0.05 CI [0.04, 0.06]	0.93	0.93	5.77(1)	*p* < 0.01

The goodness-of-fit indices for the best model are the following: χ^2^/df = 1.21, RMSEA = 0.05, confidence interval [0.04, 0.06], NNFI = 0.93, CFI = 0.93.

An evaluation of these indices in relation to the results of the analysis for this study reveals good fit and support for the PS&RM model.

## Discussion

The aim of the present study was to provide empirical support for the conceptualization of PS&RM (Di Fabio, 2014b, 2015a). The study sought to establish the empirical validity of the latent construct PS&RM operationalized by three lower order latent constructs: Positive Lifelong Life Management, Positive Lifelong Self-Management, and Positive Lifelong Relational Management.

Structural equation modeling confirmed the hypothesized structure articulated by the three lower order latent constructs: Positive Lifelong Life Management, Positive Lifelong Self-Management, and Positive Lifelong Relational Management. The construct of Positive Lifelong Life Management integrates hedonic and eudaimonic well-being, meaning in life and authenticity. Given that rapid social, economic, technological, and career change can disrupt a sense of meaning, coherence, and well-being (Masten, 2014), the capacity for sustaining well-being, meaning, and authenticity in the context of change and disruption are considered adaptive. Positive Lifelong Life Management incorporates hedonic well-being, including the presence of positive affect, the absence of negative affect (Watson et al., 1988) and the presence of life satisfaction (Diener et al., 1985). It also encompasses dimensions of eudaimonic well-being hypothesized as fundamental for optimal life management. These dimensions are aligned with prior research on the realization of authenticity (Wood et al., 2008), the authentic self (Di Fabio, 2014f), meaning in life (Morgan and Farsides, 2009), authentic meaning (Bernaud, 2015), and purposeful identitarian awareness (Di Fabio, 2014f). Di Fabio (2014a,e) recently introduced the construct of purposeful identitarian awareness as a preventive life-management competency that includes awareness of the authentic self, including knowledge of one's intrinsic interests (Sheldon and Houser-Marko, 2001) and a striving toward goals that matter both personally and socially (Di Fabio, 2014d).

The second construct Positive Lifelong Self-Management emphasizes self and intraindividual resources considered important for coping proactively with the challenges of the post-modern era (Di Fabio, 2014c). These resources encompass the meta-competencies of ISC, adaptability, and reflexivity (Di Fabio, 2014f). Adaptability resources, including concern about the future; control or the application of self-discipline, effort, and persistence to shape and prepare for the future; curiosity or the capacity to think about oneself in a variety of future roles; and confidence that one can pursue hoped-for choices and aspirations, can be drawn upon to cope with undefined, unfamiliar, and uncertain life demands (Savickas and Porfeli, 2012). Reflexivity (Guichard, 2004, 2005; Maree, 2013) contributes to personal efforts to construct personal meaning, identification of the essence of one's authentic self, and development of one's own purposeful identitarian awareness (Di Fabio, 2014f).

The third construct Positive Lifelong Relational Management includes personal (e.g., trait emotional intelligence; Petrides and Furnham, 2004) and social resources (perceived social support; Zimet et al., 1988) and the dialectic of self in relationship (positive relational management; Di Fabio, 2015c) hypothesized as central for adaptive relational functioning while managing the life challenges of the twenty-first century. The contributions of emotional intelligence and social support for adaptive social, emotional and career functioning have been widely documented in prior research (Di Fabio and Kenny, 2012a,b; Di Fabio et al., 2014). The value of positive relational management has been confirmed in studies with Italian university students and Italian workers (Di Fabio, 2016a,b), showing associations between positive relational management and acceptance of change, perceived employability, well-being, and academic and workplace relational civility in terms of relational readiness, relational culture, and relational decency toward the others.

In sum, the current study provides empirical support for the conceptualization of PS&RM as Positive Lifelong Life Management, Positive Lifelong Self-Management, Positive Lifelong Relational Management. Considered collectively, the three dimensions consider the importance of advancing a state of well-being that benefits the individual and society and promotes personal skills to adapt to change and uncertainty while integrating and balancing individual, relational, and community concerns and interests. Together, the dimensions are supportive of decent work and decent life.

Although the results of the present study test the PS&RM model (Di Fabio, 2014b, 2015a), some limitations need to be considered. The sample characteristics limit generalizability of the findings. In fact, the present research included a group of Italian university students who were not representative of Italian context. Future studies should include participants more representative of the broader Italian population, taking into consideration other geographical areas. Future studies should test the model and replicate the findings with larger samples. Future research will also need to test the PS&RM model in other international contexts and with samples across varied social classes and employment statuses. Since our goal is to develop and test an inclusive construct, establishing the construct validity of the model for diverse populations will be vital. Although the constructs and measures incorporated into the model have been validated in prior research as predictive of social, psychological and career adaptation and life-long well-being, the value of this model and its parsimony in explaining positive adaptation and well-being in comparison with alternative models needs to be established, especially with diverse populations. While our model is specified as lifespan relevant, future research should assess its applicability and value in the context of specific life transitions.

If the PS&RM model is tested and validated in future research with diverse populations, it may offer a useful framework for preventive intervention. As a preventive framework (Hage et al., 2007; Kenny and Hage, 2009; Di Fabio et al., in press), PS&RM underlines the importance of building individual (Di Fabio and Blustein, 2010; Di Fabio and Kenny, 2012a; Di Fabio and Palazzeschi, 2012; Di Fabio et al., 2012, 2013, 2014; Di Fabio and Saklofske, 2014a,b) and relational strengths (Blustein, 2011a; Di Fabio and Kenny, 2012b) that should be helpful in adapting and thriving despite the disruptions of the twenty-first century. The focus of the model on building strengths draws from positive psychology (Seligman and Csikszentmihalyi, 2000; Seligman, 2002) and emphasizes the attainment of higher levels of eudaimonic well-being, including meaning, authenticity and social purpose. Since social and economic change can be disruptive to a sense of meaning and coherence, maintaining well-being is an adaptive capacity. The PS&RM model also highlights the dialectic of the self in relationship (Blustein, 2011a; Di Fabio, 2014b, 2015a) as relevant for creating a rich career and life project (Di Fabio, 2014c) and for decent work, simultaneously balancing and integrating needs for power, for relationship, and for self-determination (Blustein, 2006). The capacity for building strengths and resources aligned with this model through preventive intervention is already being documented by initiatives of the International Laboratory of Research and Intervention in Vocational Guidance and Career Counseling and the International Laboratory in Positive Psychology and Prevention at the University of Florence (Di Fabio et al., in press). The Laboratory is engaged in the design and evaluation of school and workplace based interventions to build self and career management through the narrative assessment of career competencies, life design activities, and the promotion of specific competencies, such as emotional intelligence and intrapreneurial self-capital. The further validation of this model will provide a strong conceptual foundation for furthering these and other efforts to build culturally and contextually sensitive interventions.

It is our hope that the preventive and relational framework specified by the PS&RM model can contribute to building decent work and decent lives for many people in the twenty-first century. We propose that life construction (Guichard, 2013b) should seek to expand well-being with a focus on positive life management, positive self-management and positive relational management. PS&RM might thus be a framework for building well-being and psychological decency through attention to the authentic self (Di Fabio, 2014f), purposeful identitarian awareness (Guichard, 2013b; Di Fabio, 2014f), and the centrality of relational processes (Blustein, 2011a). It is essential to recognize that the model is entirely psychological and thus limited in its capacity to foster decent work and decent lives. We focus in this model on the subjective experience of decent work and life and what vocational and relational psychology can do to assist persons in developing the resources to thrive despite challenging and disruptive life circumstances. In recognition that this state of uncertainty and disruption may be persistent, we emphasize the value of the individual's psychological resources in positively and constructively managing one's self, life, and career. Nonetheless we are also hesitant to place too much responsibility for adaption and well-being on individuals. In a multidisciplinary framework, the psychological perspective complements the perspectives of economics and public policy in framing interventions to promote decent work. The psychological perspective does not, however, preclude the economic, social, organizational, and political policies and actions that need to be implemented to broaden opportunity and access to resources, so that equal access to decent work, rather than adjustment to scarcity, insecurity, uncertainty, and inequality, is the context for life construction.

## Author contributions

AD and MK conceptualized the study, chose the theoretical framework and chose the measures. AD collected the data and with MK wrote the methods and results. Then the authors wrote the paper together and read and revised the manuscript several times.

### Conflict of interest statement

The authors declare that the research was conducted in the absence of any commercial or financial relationships that could be construed as a potential conflict of interest.
